# T-lymphokine-activated killer cell-originated protein kinase (TOPK) as a prognostic factor and a potential therapeutic target in glioma

**DOI:** 10.18632/oncotarget.23674

**Published:** 2017-12-26

**Authors:** Chuntao Quan, Juanjuan Xiao, Qiuhong Duan, Ping Yuan, Peipei Xue, Hui Lu, Meng Yan, Dongsheng Guo, Sanpeng Xu, Xiaohui Zhang, Xuan Lin, Yong Wang, Soner Dogan, Jianmin Zhang, Feng Zhu, Changshu Ke, Lin Liu

**Affiliations:** ^1^ Department of Biochemistry and Molecular Biology, School of Basic Medicine, Huazhong University of Science and Technology, Wuhan, Hubei, PR China; ^2^ Department of Pathology, Affiliated Tianyou Hospital of Wuhan University of Science and Technology, Wuhan, Hubei, PR China; ^3^ Department of Neurosurgery, Tongji Hospital, Tongji Medical College, Huazhong University of Science and Technology, Wuhan, Hubei, PR China; ^4^ Department of Pathology, Tongji Hospital, Tongji Medical College, Huazhong University of Science and Technology, Wuhan, Hubei, PR China; ^5^ Department of Hematopathology and Laboratory Medicine, H. Lee Moffitt Cancer Center and Research Institute, Tampa, Florida, USA; ^6^ Department of Endocrinology, China Resources and WISCO General Hospital, Wuhan, Hubei, PR China; ^7^ School of Medicine, Yichun University, Yuanzhou District, Yichun, Jiangxi, PR China; ^8^ School of Medicine, Yeditepe University, Istanbul, Turkey

**Keywords:** glioma, TOPK, prognostic factor, TMZ, drug resistance

## Abstract

TOPK is overexpressed in various types of cancer and associated with poor outcomes in different types of cancer. In this study, we first found that the expression of T-lymphokine-activated killer cell-originated protein kinase (TOPK) was significantly higher in Grade III or Grade IV than that in Grade II in glioma (*P* = 0.007 and *P* < 0.001, respectively). Expression of TOPK was positively correlated with Ki67 (*P* < 0.001). Knockdown of TOPK significantly inhibited cell growth, colony formation and increased sensitivities to temozolomide (TMZ) in U-87 MG or U-251 cells, while TOPK overexpression promoted cell growth and colony formation in Hs 683 or A-172 cells. Glioma patients expressing high levels of TOPK have poor survival compared with those expressing low levels of TOPK in high-grade or low-grade gliomas (hazard ratio = 0.2995; 95% CI, 0.1262 to 0.7108; *P* = 0.0063 and hazard ratio = 0.1509; 95% CI, 0.05928 to 0.3842; *P* < 0.0001, respectively). The level of TOPK was low in TMZ-sensitive patients compared with TMZ-resistant patients (*P* = 0.0056). In TMZ-resistant population, patients expressing high TOPK have two months’ shorter survival time than those expressing low TOPK. Our findings demonstrated that TOPK might represent as a promising prognostic and predictive factor and potential therapeutic target for glioma.

## INTRODUCTION

Glioma represents the most common primary tumor in the Central Nervous System (CNS). Along with the increased incidence of brain tumors, glioma mortality in China increased by as much as 194% in 2008, compared to data from the 1970s [1]. Malignant glioma often leads to fatal outcomes because of its biological behavior and its resistance to current therapies, posing great challenges to public health. For the past century, the classification of glioma has been mostly based on histopathological features. However, some patients with histologically identical tumors have very different outcomes and responses to treatment. Integration of both histological and molecular biomarkers was introduced in the 2016 World Health Organization (WHO) Classification of CNS tumors [2]. Therefore, it is critical to identify important candidates to improve our understanding on the pathogenesis, diagnosis, clinical therapeutic decision and prognosis evaluation in malignant glioma patients.

T-lymphokine-activated killer cell-originated protein kinase (TOPK) is a MAPKK-like serine/threonine protein kinase extensively expressed in various types of cancer, such as colorectal cancer [3], lymphoma [4], melanoma [5], breast cancer [6], lung cancer [7], and cholangiocarcinoma [8]. Previous studies indicated that TOPK is involved in many important biological processes including mitosis [9], cell proliferation [10], DNA repair [11], and carcinogenesis [3]. Additionally, levels of TOPK could be closely associated with prognostic diagnosis in colorectal cancer [12], lung cancer [13], and ovarian cancer [14].

Temozolomide (TMZ), a first line chemotherapeutic drug for glioma patients [15], can induce DNA lesions including N^7^-MeG, N^3^-MeA and O^6^-MeG by DNA methylation, leading to DNA double strand breaks, thereby exerting its anti-cancer cytotoxicity [16]. As a predominant DNA lesion produced by TMZ, O^6^-MeG, is repaired by O^6^-methylguanine-DNA methyltransferase (MGMT), which is a DNA repair protein [17]. The level of MGMT expression is increased when the *MGMT* gene promoter is unmethylated, which enhances tumor resistance to TMZ [18]. In addition to MGMT repair mechanism, mismatch repair [19] and base excision repair [20] are involved in TMZ resistance as well.

In this study, we found that TOPK expression was significantly increased in high-grade gliomas (HGG) (WHO Grade III & IV) patients. TOPK expression was closely associated with glioma grading, poor survival of glioma patients, cell proliferation and tumorigenesis of glioma, and more importantly, with chemotherapeutic resistance to TMZ.

## RESULTS

### TOPK is overexpressed in HGG patients

**Figure 1:**
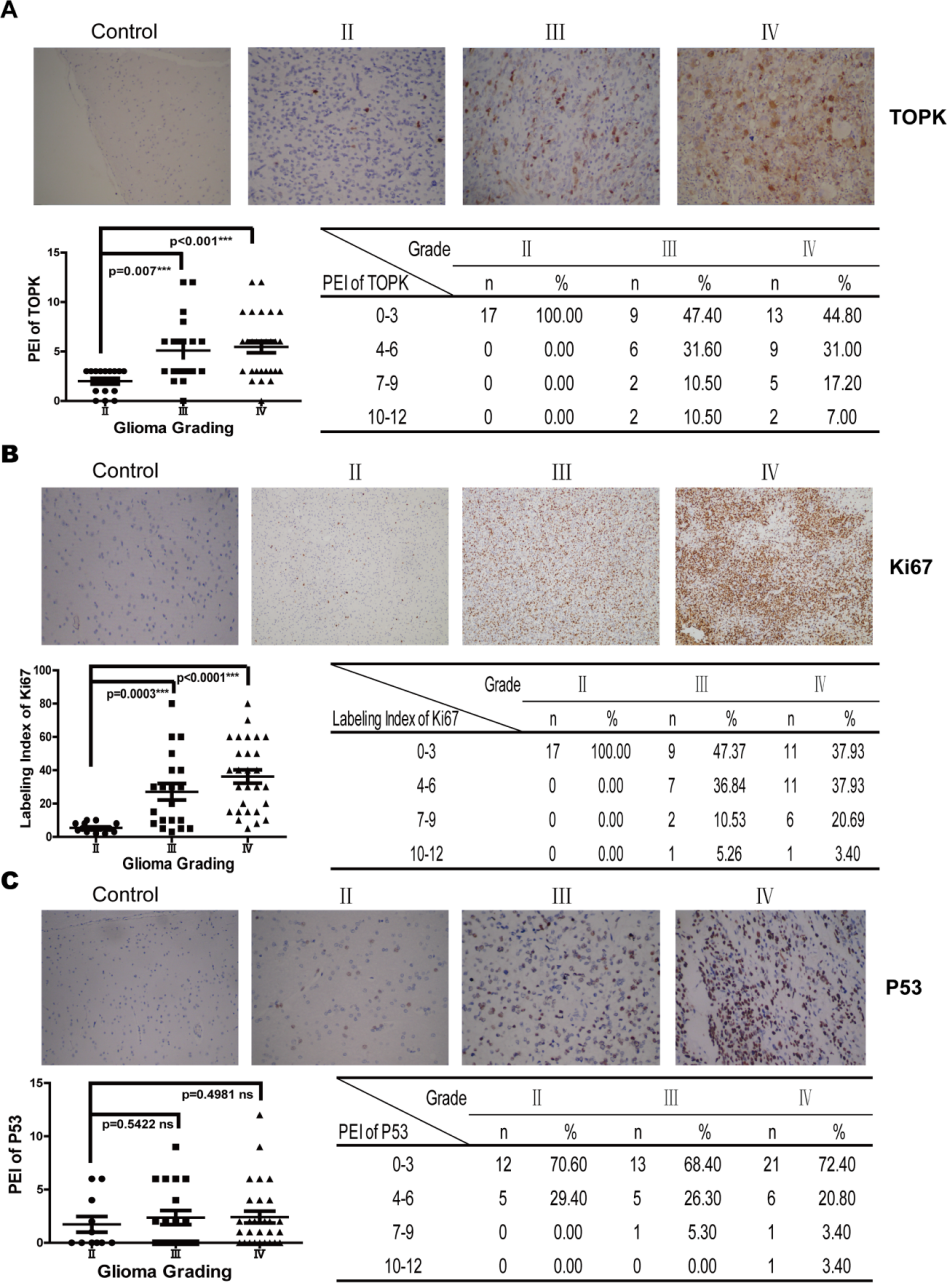

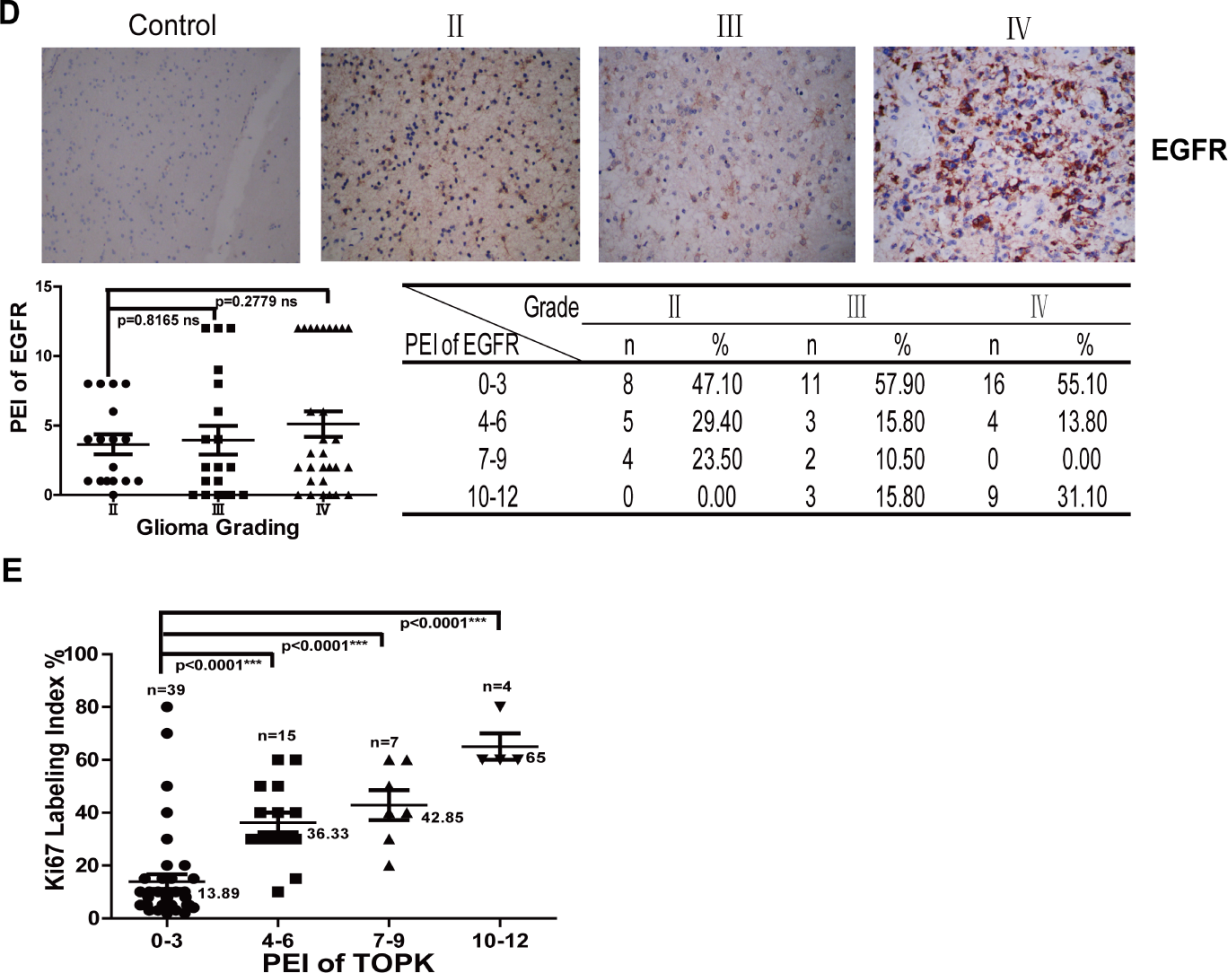
TOPK is overexpressed in HGG patients (**A**) IHC examination of TOPK expression in tissues from 65 cases of human glioma. Pictures from one representative case are shown in the *upper panel*. Statistics of the IHC examination results are shown in *lower panel*. The asterisk indicates a significant increase in TOPK expression in Grade III or Grade IV compared with Grade II (*P* = 0.007 and *P* < 0.001, respectively). Expression of Ki67 (**B**), P53 (**C**) and EGFR (**D**) was examined by IHC in the same samples. The pictures presented are representative images of IHC staining (*upper panel*) and statistics of the IHC examination results are shown in *lower panel* (**E**) The correlation between protein expression of TOPK and Ki67 was analyzed.

The expression level of TOPK was analyzed in low-grade gliomas (LGG) (Grade II, 17 cases) or HGG (Grade III, 19 cases; Grade IV, 29 cases) patient samples by IHC. As shown in Figure 1A, TOPK expression was negative in patients with focal cortical dysplasia, weak in Grade II, and significantly stronger in Grade III or Grade IV compared to expression in Grade II (*P* = 0.007 and *P* < 0.001, respectively). No significant difference in TOPK expression was found between Grade III and Grade IV (*P* = 0.6973). Expression levels of TOPK were scored from 0 to 12 according the definition described in the materials and methods section. High score of TOPK was only observed in HGG patients. These data suggested that TOPK was related with histological grade and could act as a promising diagnostic factor for differentiating HGG and LGG. Meanwhile, we examine the expression of Ki67, P53 and EGFR, common molecules associated with histological grade or prognosis in human glioma. Ki-67, reflecting the proliferation and malignancy of cancer cells, was significantly increased with the grade of glioma [21]. Our results also showed that Ki67 was significantly increased in Grade III or Grade IV glioma compared to Grade II glioma (*P* = 0.0003 and *P* < 0.0001, respectively) (Figure 1B). EGFR gene amplification is one of the most frequent genetic alterations observed in glioma [22], and EGFR expression generally correlates with WHO grade in gliomas [21]. P53 mutations were found in glioblastomas, astrocytomas and anaplastic astrocytomas [23]. Studies reported that mutant P53 was positively correlated with TOPK expression in cancer cell [7]. We found that P53 and EGFR were expressed in LGG or HGG, and no significant difference between Grade II and Grade III or Grade IV (*P* > 0.05) (Figure 1C and 1D). Furthermore, our results demonstrated that significant correlation was identified only between TOPK and Ki67 expression (*P* < 0.0001) (Figure 1E), not between TOPK and EGFR or P53 (data not shown).

### Knockdown of TOPK reduces tumorigenic properties *ex vivo*

**Figure 2:**
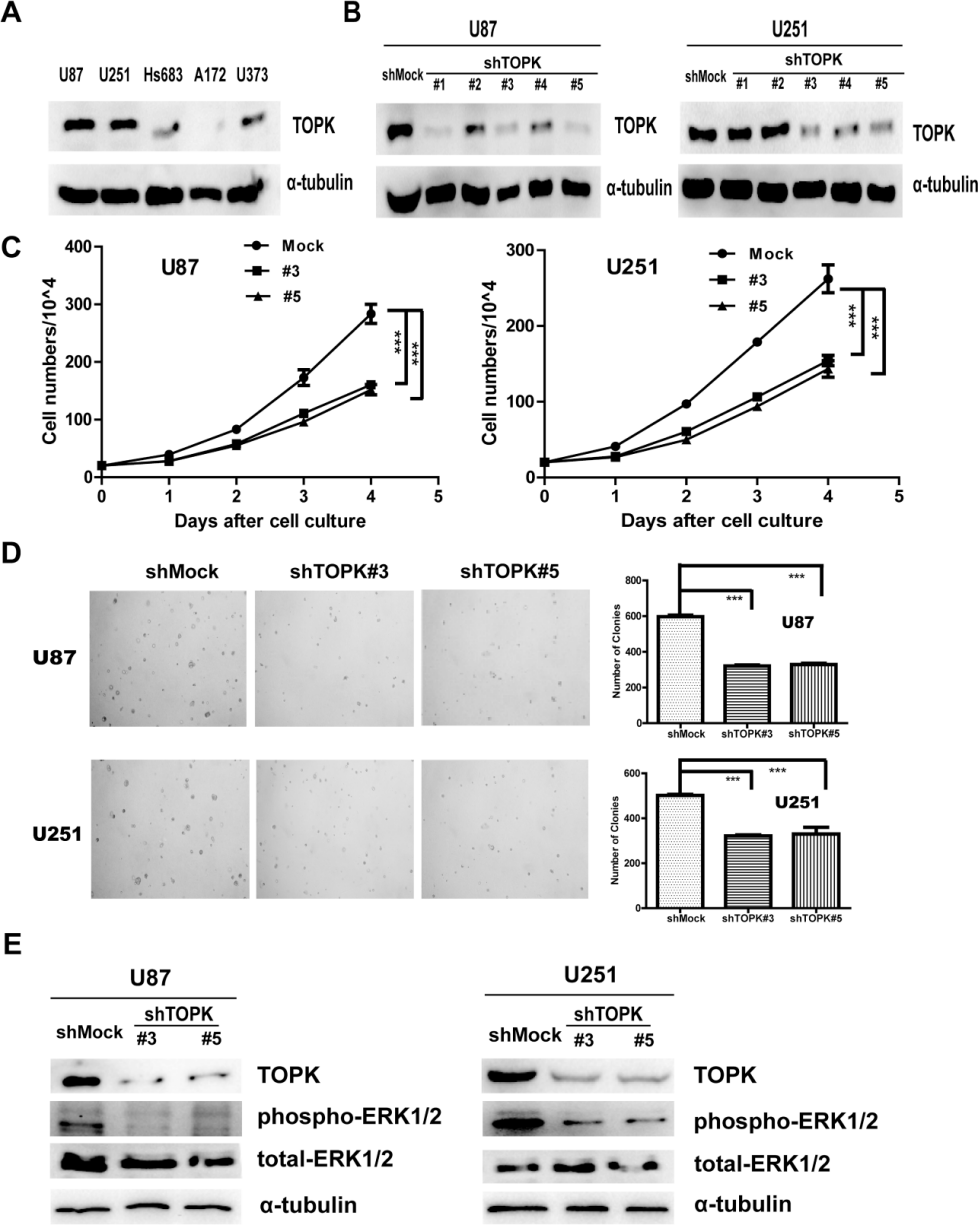
Knockdown of TOPK reduces tumorigenic properties *ex vivo* (**A**) Expression of TOPK was determined in five glioma cell lines by Western blot. (**B**) TOPK expression in TOPK knockdown cell lines was identified by Western blot. (**C**) Growth curves of U-87 MG/shMock, U-87 MG/shTOPK#3 and U-87 MG/ shTOPK#5 (*left panel*) or U-251/shMock, U-251/shTOPK#3 and U-251/shTOPK#5 (*right panel*). Data are presented as mean ± standard deviation from triplicate experiments. The asterisk indicates a significant decrease in cell number in shTOPK cells compared with shMock cells. (**D**) Colony formation by U-87 MG/shMock, U-87 MG/shTOPK #3 or U-87 MG/shTOPK #5 transfectants were compared in soft agar. The asterisk indicates a significant decrease in cell number in shTOPK cells compared with shMock cells. Similar results were observed in the U-251/shMock or U-251/shTOPK cell lines. (**E**) The level of phospho-ERK1/2 was detected in shMock cells and shTOPK cells.

It has been reported that TOPK could promote tumorigenesis of colorectal cancer [3]. As shown above, TOPK was highly expressed in HGG, therefore we attempted to assess the role of TOPK in glioma using glioma cell models. First, the level of endogenous TOPK was tested in 5 different human glioma cell lines. Results showed that levels of TOPK in U-87 MG or U-251 cells were higher than those in A-172, Hs 683 or U373 cells (Figure 2A). Therefore, we used shRNAs to knock down TOPK in U-87 MG or U-251 cells (U-87 MG/shTOPK or U-251/shTOPK) and U-87 MG/shMock or U-251/shMock is control. As shown in Figure 2B, TOPK expression was knocked down by shRNA sequence #3 and #5 in both cells. Next, growth curves of U-87 MG/shMock, U-87 MG/shTOPK#3 or U-87 MG/shTOPK#5 cells were compared, and the results indicated that U-87MG/shTOPK cells grew dramatically slower than U-87 MG/shMock cells (Figure 2C *left panel*). Next, the anchorage-independent growth of the U-87 MG/shMock or U-87 MG/shTOPK cell lines was examined, and the results indicated that the number of colonies in U-87 MG/shTOPK cell cultures was much less than that in U-87 MG/shMock cell cultures (Figure 2D *upper panel*). Similar results were observed in the U-251/shMock or U-251/shTOPK cell lines (Figure 2C *right panel* and Figure 2D *lower panel*). ERK has been identified as one of the substrates of TOPK and TOPK-ERK interaction increases tumorigenesis of colorectal cancer cells [3]. Next, we assessed the level of phospho-ERK1/2 in shMock cells and shTOPK cells. Decreased phospho-ERK1/2 was observed in shTOPK cells (Figure 2E). Therefore, these results indicated that suppression of TOPK in glioma cells inhibited tumorigenesis *ex vivo*.

### TOPK overexpression promotes tumorigenic properties *ex vivo*

**Figure 3:**
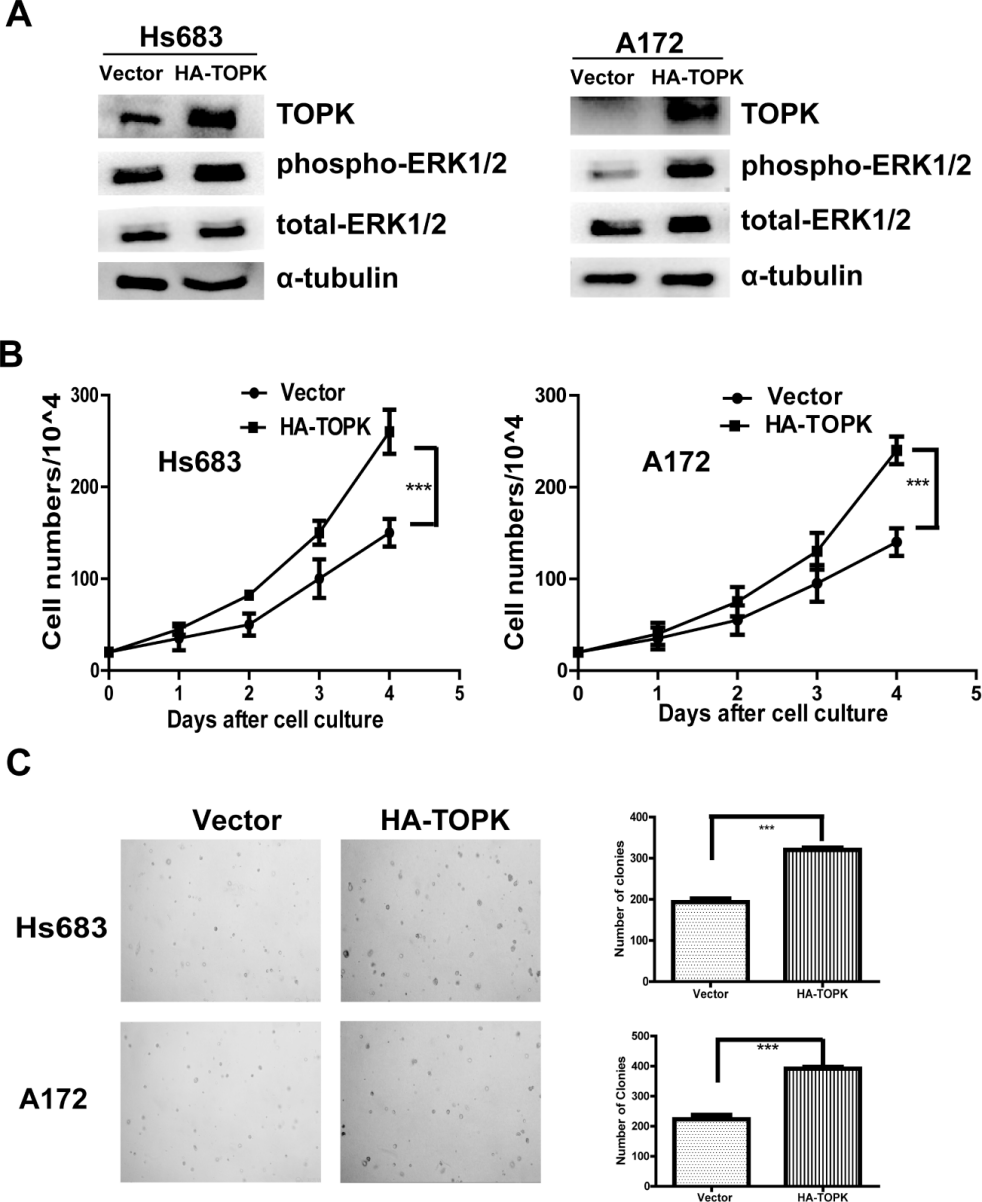
TOPK overexpression promotes tumorigenic properties *ex vivo* (**A**) Expression of TOPK and phospho-ERK1/2 in Hs 683 or A-172 cells after transfection with pcDNA3 or pcDNA3-HA-TOPK. (**B**) Growth curves of Hs 683/vector and Hs 683/TOPK (*left panel*) or A-172/vector and A-172/TOPK (*right panel*). Data are presented as mean ± standard deviation from triplicate experiments. The asterisk indicates a significant increase in cell number in Hs 683/TOPK or A-172/TOPK cells compared with Hs 683/vector or A-172/vector control cells. (**C**) Colony formation in soft agar of vector control cells (Hs 683/vector) compared with TOPK-overexpressing cells (Hs 683/TOPK) is shown (*upper left panel*). Similar experiment using A-172/vector and A-172/TOPK cells (*lower left panel*). The asterisk indicates a significant increase in cell number in TOPK-overexpressing cells compared with control cells.

In order to further confirm the role of TOPK on glioma cell growth, we transfected pcDNA3 or pcDNA3-HA-TOPK into Hs 683 or A-172 cells to generate TOPK overexpression cell lines. Expression of TOPK and phospho-ERK1/2 was increased (Figure 3A). Cell proliferation (Figure 3B) and colony formation (Figure 3C) were significantly increased upon TOPK overexpression in Hs 683 or A-172 cells. These results demonstrate that TOPK promotes tumorigenic effects *ex vivo*.

### TOPK inhibits TMZ-induced apoptosis in U-87 MG and U-251 cells

**Figure 4:**
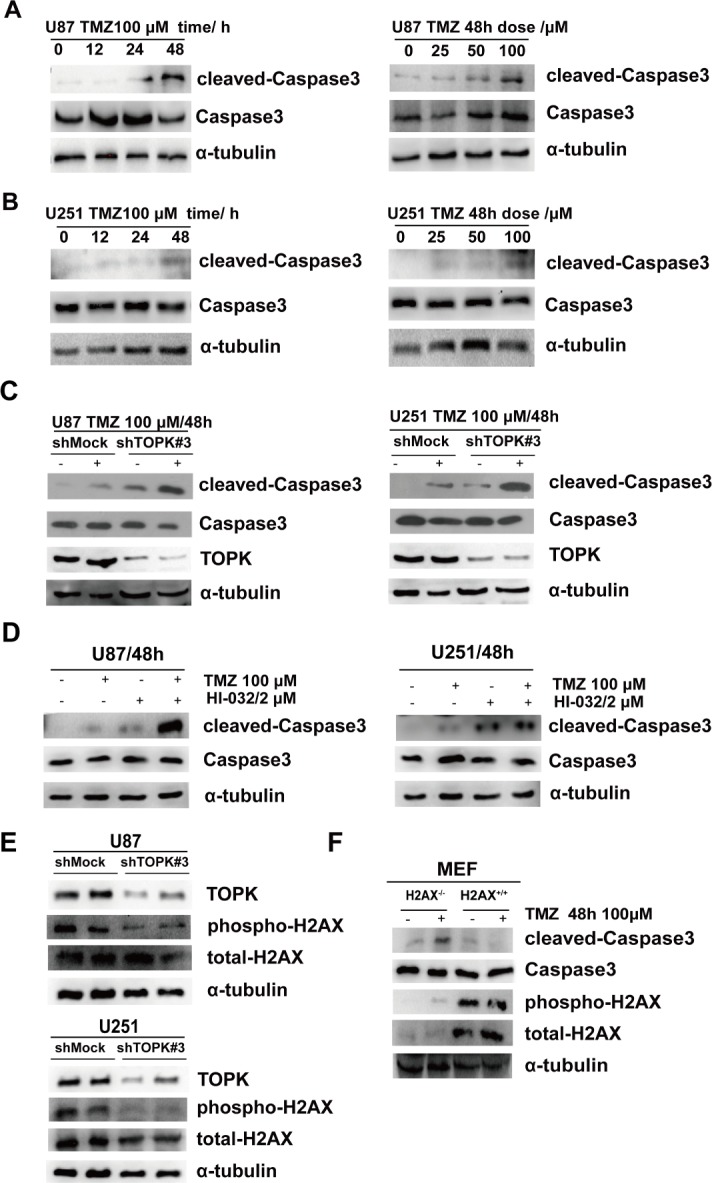
TOPK inhibits TMZ induced apoptosis in U-87 MG and U-251 cells TMZ induced a time- and dose-dependent expression of cleaved-Caspase3 in U-87 MG cells (**A**) or U-251 cells (**B**). (**C**) The expression of cleaved-Caspase3 was analyzed in U-87/shTOPK cells (*left panel*) or and U-251/shTOPK cells (*right panel*) treated with TMZ (48 h /100 μM) by Western blot. (**D**) The expression of cleaved-Caspase3 was analyzed in U-87 MG cells (*left panel*) or U-125 cells (*right panel*) treated with TMZ or/and HI-032 by Western blot. (**E**) The expression of γ-H2AX was analyzed in U-87/shTOPK cells (*upper panel*) or U-251/shTOPK cells (*lower panel*) by Western blot. (**F**) The levels of γ-H2AX and cleaved-Caspase3 was analyzed in H2AX^+/+^ and H2AX^−/−^ MEF cells treated with TMZ by Western blot.

TMZ is a first-line chemotherapeutic drug for clinical glioma therapy [16]. However, tumor cells can develop resistance to TMZ, diminishing its therapeutic effects. The above data indicated that TOPK was very important in glioma development, therefore we decided to test whether TOPK expression is related to TMZ resistance in glioma. First, we treated U-87 MG or U-251 cells with TMZ in a time- or dose- dependent manner. The level of cleaved-Caspase3 gradually increased and reached its highest level after 48 h treatment with 100 μM TMZ (Figure 4A, 4B). Next, we treated shMock or shTOPK-supplemented U-87 MG or U-251 cells with 100 μM TMZ for 48 h and assessed whether endogenous TOPK affect TMZ-mediated caspase activation. We found that shTOPK-treated cells displayed an increase in cleaved-Caspase3 compared with shMock-treated cells when treated with TMZ, which demonstrated endogenous TOPK inhibited TMZ-mediated caspase activation and cell apoptosis (Figure 4C). Further demonstrating the effect of TOPK on glioma cells apoptosis in present of TMZ, we treated cells with HI-032, an inhibitor of TOPK [24]. The concentration of HI-032 was determined following IC50 value of HI-032 for U87 and U251 cells (data not shown) and related references [24]. Results indicated that cleaved-Caspase3 was increased in U-87 MG or U-251 cells treated for 48 h with 100 μM TMZ and 2 μM HI-032 compared to cells treated with TMZ alone (Figure 4D). These findings suggest that TOPK suppression in glioma cells could promote cell apoptosis and enhance TMZ sensitivity. TMZ can induce DNA double strand breaks (DSBs) [25]. Serine 139 phosphorylation of histone H2AX (γ-H2AX) is an important sensor for this kind of DNA damage [26]. Next, the expression of γ-H2AX was examined in shMock or shTOPK cells. The results showed that γ-H2AX was significantly down-regulated in shTOPK cells (Figure 4E). H2AX^+/+^ MEF and H2AX^−/−^ MEF cells were treated with TMZ, and we observed that knockout of H2AX promoted cell apoptosis (Figure 4F). All these data indicated that TOPK blocked TMZ-induced glioma cell apoptosis through increasing γ-H2AX.

### TOPK is a prognostic and predictive factor for glioma

**Figure 5:**
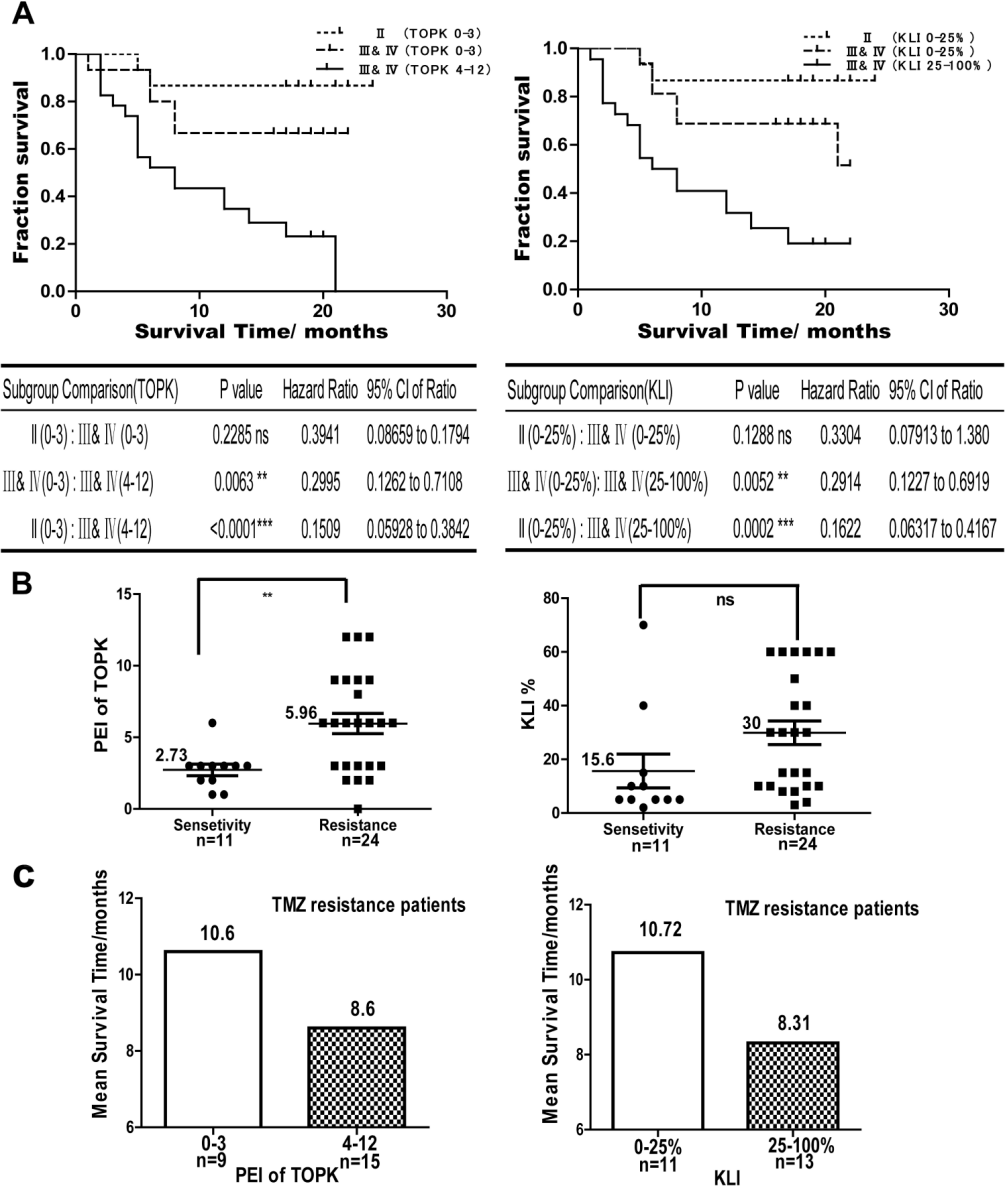
TOPK is a prognostic and predictive factor for glioma (**A**) Log-rank test showing the correlation between the level of TOPK (*left panel*) or Ki67 and survival (*right panel*). (**B**) The correlation between the level of TOPK (*left panel*) or Ki67 (*right panel*) and TMZ resistance was assessed by Student's *t*-test. The asterisk indicates a significant correlation between TMZ resistance and TOPK expression (*P* = 0.0056). (**C**) In the TMZ-resistant patient population, the correlation between survival time and levels of TOPK (*left panel*) or Ki67 (*right panel*) were analyzed.

Since TOPK can block TMZ-induced glioma cell apoptosis, we next investigated the correlation between the level of TOPK and patient survival and the relationship between TOPK expression level and TMZ treatment. We followed up 57 cases glioma patients and divided them into two groups, a low TOPK expression group (score: 0–3) and a high TOPK expression group (score: 4–12). Patients with high TOPK expression had a shorter median survival time (MST) and overall survival (OS) than patients with low TOPK expression. Log-rank test showed that glioma patients with high levels of TOPK have poor survival outcome compared with those with low levels of TOPK in HGG or LGG (hazard ratio = 0.2995; 95% CI, 0.1262 to 0.7108; *P* = 0.0063 and hazard ratio = 0.1509; 95% CI, 0.05928 to 0.3842; *P* < 0.0001, respectively). HGG and LGG patients with low TOPK expression has no significant difference for OS (hazard ratio = 0.3941; 95% CI, 0.08659 to 0.1794; *P* = 0.2285) (Figure 5A *left panel*). These results showed that TOPK overexpression obviously shortens the length of OS in glioma, and TOPK is correlated with poor survival outcome, regardless of WHO Grade. We also compared the correlation between Ki67 expression and survival in the same patient samples, and the results indicated that patients with high Ki67 expression have poor survival outcome as well. (hazard ratio = 0.2914; 95% CI, 0.1227 to 0.6919; *P* = 0.0052; and hazard ratio = 0.1622; 95% CI, 0.06317 to 0.4167; *P* = 0.0002) (Figure 5A *right panel*). Therefore, we hypothesize that TOPK can act as a prognostic factor similarly to Ki67 in glioma. To identify the potential association between TOPK expression and the efficacy of TMZ chemotherapy, 35 patients received TMZ chemotherapy were studied. The results indicated that patients with high levels of TOPK were insensitive to TMZ therapy compared to those with low levels of TOPK (*P* = 0.0056) (Figure 5B *left panel*), which suggested that TOPK expression was significantly related to TMZ resistance. No significant correlation between Ki67 and TMZ treatment was identified (*P* = 0.075) (Figure 5B right panel). Furthermore, we analyzed the correlation between TOPK or Ki67 expression level and survival in the 24 cases of TMZ-resistant patients. The results indicated that patients with high levels of TOPK or Ki67 have at least two months’ shorter survival time than those with low level of TOPK or Ki67 in TMZ-resistant patients (Figure 5C). Therefore, TOPK is a useful prognostic and predictive factor in glioma patients.

## DISCUSSION

The WHO redefined the classification of CNS tumors in 2016. Unlike the previous editions of WHO classification based on histopathological features, through integrating histopathological and molecular features in glioma diagnosis, more prognostic information is provided. The most frequent genetic alterations associated with glioma initiation and progression include mutation of *isocitrate dehydrogenase* 1/2 (*IDH1/2*) [27], co-deletion of chromosomes 1p and 19q (1p/19q co-deletion) [28], *p53* mutation [21] and *EGFR* amplification or mutation [21, 29]. These molecular alterations are greatly helpful to supplement the histological evaluation in defining specific glioma subgroups. However, WHO grade identifications are still based on histopathological features, more efforts are trying to identify prominent molecular markers representing the progression status, i.e. the grades of the glioma. Recently, some new biomarkers have been identified to be correlated with glioma grading [30, 31]. In the present study, we demonstrated for the first time that TOPK expression was increased in glioma, and the level of TOPK was significantly higher in Grade III or Grade IV than that in Grade II. Meanwhile, we examined the expression of Ki67, P53, EGFR in glioma patients with different grade, and association between glioma grading and Ki67 or P53 was the same with what have been reported. While the level of EGFR had no significant correlation with glioma grading, which was inconsistent with prior findings. The EGFR overexpression was mostly found in HGG [21, 29], because of the limitation of numbers of LGG, as a whole, the association of EGFR expression with grading might not be appreciated in the current study. We also found that TOPK was positively correlated with Ki67 expression. Ki67 is associated with histological grade and poor survival outcomes in glioma cases [21, 32], suggesting the level of TOPK is significantly related to glioma grade progression, and TOPK act as a novel diagnostic factor to identify HGG and LGG patients.

TOPK contribute to tumorigenesis, tumor development and progression [3, 33–38], TOPK was reported to promote tumor development and progression through suppression of P53 function in HCT116 cells [39]. TOPK increased cell migration by modulating PI3K/PTEN/AKT-dependent signaling pathway in lung cancer [40]. High TOPK expression is associated with poor outcome, and TOPK has been identified as prognostic marker in various cancer [12–14]. Lung adenocarcinoma patients with high level of TOPK expression had shorter OS and time to recurrence when compared to patients with lower level of TOPK expression [41]. High level of TOPK expression was associated with poor progression-free survival (PFS) and OS in ovarian cancer [14]. So far, little research has been reported about the association between TOPK expression and patient survival in glioma. Our results showed that TOPK promoted tumorigenic properties and glioma cellular proliferation in glioma cells. Glioma patients with high levels of TOPK have poor survival outcome compared with those with low levels of TOPK in HGG or LGG, and patients with low TOPK expression, whether HGG or LGG, had good survival outcome, which suggests that TOPK serves as a prognostic factor regardless of WHO grade in glioma. Therefore, TOPK could promote glioma malignant potential and progression and contribute to poor prognosis for glioma patients.

Currently, the standard treatment strategies for glioma are surgical resection, radiotherapy and adjuvant chemotherapy. TMZ is a commonly preferred chemotherapy drug for glioma patients. MGMT was identified as an important biomarker capable of predicting the sensitivity of glioma patients to TMZ treatment. 50% of glioblastomas patients with low level of MGMT could not benefit from TMZ therapy, which suggested that the additional factors participated in TMZ resistance [42]. In this study, clinical results demonstrated that patients with high levels of TOPK have worse survival outcomes than those with low levels of TOPK in TMZ-resistant patients. This result indicated that high levels of TOPK were associated with TMZ resistance. Studies reported that TOPK could activate NF-κB in HeLa cells [43] and reduction of NF-κB activity could reverse TMZ resistance by MGMT expression in glioma cells [44], which implied that TOPK could promote MGMT expression through activation of NF-κB to lead to TMZ resistance. In the current study, we found that blocking TOPK expression promoted glioma cell apoptosis when treatment with TMZ in U-87 MG and U-251 cells. *MGMT* gene mRNA was barely detectable in both U-87 MG and U-251 cells [45]. We also could not detect protein of MGMT in U-87 MG cell, a cell line with highly endogenous TOPK expression (data not shown). Therefore, we thought that TOPK promoting TMZ resistance was probably through other mechanism besides MGMT mechanism in glioma cells. TOPK could regulate DNA repair [11]. TOPK could phosphorylate H2AX directly [46]. We found that blocking TOPK decreased γ-H2AX expression in U-87 MG and U-251 cells. γ-H2AX is required for the accumulation of DNA damage response (DDR) proteins and the defects of DNA damage repair response associated with downregulation of TOPK expression sensitized cells to TMZ treatment. Overall, our results demonstrated that TOPK expression was available to predict TMZ-sensitive and TMZ-resistant glioma patients. However, whether TOPK reducing TMZ sensitivity is associated with MGMT expression requires further investigation.

TMZ treatment has improved PFS and OS in glioma patients, but its survival benefit remains unsatisfactory because of primary or acquired resistance. In this study, we demonstrated that TOPK was highly expressed in HGG, and high level of TOPK was significantly associated with TMZ resistance and poor survival in glioma patients. TOPK promotes glioma cellular proliferation and tumorigenesis. Either HI-032 treatment or TOPK knockdown can promote cell apoptosis to TMZ treatment. It has been reported that targeting TOPK decreased growth and survival of glioma initiating cells *in vitro* and inhibited tumor growth *in vivo* [10]. Therefore, TOPK may serve as a potential therapeutic target for TMZ treated glioma patients, especially for HGG patients. Recently, several TOPK inhibitors including Caffeic acid [47], HI-032 [21], OTS964 [48] and pantoprazole [33] have been identified. Thus, combining TMZ treatment with a TOPK inhibitor may represent a promising therapy in malignant glioma.

In conclusion, our study demonstrates that TOPK is highly expressed in HGG and contributes to TMZ resistance and poor survival in glioma patients. In addition, our results show that TOPK facilitates glioma cellular growth and tumorigenesis. Therefore, TOPK can serve as a promising new diagnostic, prognostic and predictive factor and a potential therapeutic target for glioma.

## MATERIALS AND METHODS

### Clinical data

The study materials consisted of 65 cases of malignant gliomas (WHO Grade II~IV), collected from archival files dating from 2015 to 2016 of Department of Pathology, Tongji Hospital, Tongji Medical College, Huazhong University of Science and Technology. Tissue blocks from three cases of focal cortical dysplasia and one case of pilocytic astrocytoma (WHO Grade I) were designated as the control group. Clinical parameters including age, grade, MST and TMZ treatment were evaluated (See Table 1). Two pathologists reviewed the histopathological diagnosis of these malignant glioma groups simultaneously, according to the WHO Classification of Tumors of CNS.

**Table 1:** Clinical characteristics of patients

Characteristic	Total	Glioma grading		
		II	III	IV
**Sex**				
Sex ratio, M/F	1.32	1.43	0.72	1.9
Male, *n*	37	10	8	19
Female, *n*	28	7	11	10
**Age, y**				
Median	45.24	40	41.4	50.8
Range	13–74	13–56	14–63	13–74
**Survival**				
Cases of survival time, *n*	57	17	19	21
Median survival time (mo)	13.51	18.63	12.88	10
**TMZ treatment**				
Cases of sensitivity, *n*	11	5	5	1
Cases of Resistance, *n*	24	4	9	11

### Histopathology and immunohistochemistry

The tumor tissues were routinely fixed in 10% buffered formalin for at least 12 hours, and processed, embedded in paraffin block. 5 μm paraffin sections were microtomed, gradually dewaxed into the water, and the Hematoxyline & Eosin staining was performed. The histopathological features were evaluated under light microscope (Nikon 80i). For immunohistochemistry (IHC), the paraffin sections were gradually dewaxed into the water, then immersed in 0.01 M citrate buffer and microwaved for antigen retrieval. After incubation in primary antibodies against TOPK, P53, EGFR and Ki67 overnight, the standard DAKO ChemMateTM EnVision Kit, based on the two-step labeled horseradish perioxidase method, were used according to manufacturer's instructions. 3,3′-diaminobenzidine was used as the chromogen. The deposition of granular dark brown pigments in tumor cells were considered as positive staining.

### Quantification of TOPK, Ki67, P53, EGFR

The hot-spot method was used for semi-quantification of immunohistochemical expression scores. The expression of each protein was evaluated independently by two pathologists using the semi-quantitative classifications proposed by Yuan J, *et al.* [49] and Montgomery RM, *et al.* [50]. The semi-quantified positive expression indexes (PEI, from 0 to 12) of TOPK, P53 and EGFR were calculated by multiplying percentage score (percentage of positivity of tumor cells: score1 = 0–25%; score2 = 26–50%; score3 = 51–75%; and score4 = 76–100%) with staining intensity score (none = 0, weak = 1, moderate = 2, intense = 3). Ki67 labeling index was calculated as percentage of nuclear positivity of tumor cells. The PEI ≥1 were considered as positive expression.

### Plasmids, shRNA, antibodies, and other reagents

The plasmids pcDNA3-HA-TOPK and pcDNA3 were provided by our laboratory. Five shRNA sequences were designed to knock down TOPK. These sequences are: 1. 5′-CCGGGGGAACTAGGCCACCTATTAACTCGAGTTAATAGGTGGCCTAGTTCCCTTTTTG-3′; 2. 5′-CCGGGAAGTGTGGCTTGCGTAAATACTCGAGTATTTACGCAAGCCACACTTCTTTTTG-3′; 3. 5′-CC GGGTAATGATCATTATCGAAGTGCTCGAGCACTT CGATAATGATCATTACTTTTTG-3′; 4. 5′-CCGGGCC TTCATCATCCAAACATTGCTCGAGCAATGTTTGGA TGATGAAGGCTTTTTG-3′;5. 5′CCGGGATTCCACA CATTAATCTTTCCTCGAGGAAAGATTAATGTGTGG AATCTTTTTG-3′. The sense and anti-sense oligonucleo tides were synthesized, annealed and cloned into the pLKO.1-TRC cloning vector at the *EcoR I* and *AgeI* sites as described by the manufacturer [51]. A shMock was used with a sequence lacking significant homology to the human genome database. Anti-mouse TOPK and β-actin were purchased from Santa Cruz Technology, Inc. (Santa Cruz, CA, USA). Anti-H2AX, Anti-phospho-H2AX, Anti-P53, Anti-EGFR, Anti-phospho-ERK1/2 and Anti-cleaved-Caspase3 were from Cell Signaling Technology, Inc. (Boston, MA, USA). Anti-Ki67 was purchased from DAKO Company. HRP-labeled Goat anti Mouse IgG (H+L) and Goat anti Rabbit IgG (H+L) were from EarthOx, LLC (San Francisco, CA, USA). DAKO ChemMateTM EnVision Kit (horseradish peroxidase (HRP)/3,3′-diaminobenzidine (DAB), rabbit/mouse) was from Shanghai Gene Company (Shanghai, China). Simple-Fect was from Signaling Dawn Biotech (Wuhan, China). G418, puromycin, TMZ and HI-032 (an agonist binding to the active site of TOPK) were from Sigma (St. Louis, USA).

### Cell lines and culture condition

The human glioma cell lines U-87 MG, U-251, A-172, Hs 683 and the normal cell line HEK293T were purchased from American Type Culture Collection (ATCC), The cell lines were cultured in DMEM supplemented with 10% FBS at 37°C, 5% CO2 incubator following the procedures provided by ATCC and were used within 6 months of resuscitation.

### Western blot

Cells (2 × 10^6^) were seeded onto 10-cm-diameter dishes to 70–80% confluence and harvested in 200 μl RIPA buffer. 50–120 μg of protein was separated by 10%–12.5% SDS-PAGE and transferred to PVDF membranes. Then antibodies were used and antibody-bound proteins were visualized by chemiluminescence in triplicate.

### Growth curve analysis

Cells (2 × 10^5^) were cultured in 10-cm-diameter dishes and counted at different time points in triplicate, using a hemacytometer to count cell numbers and generate a growth curve.

### Anchorage-independent cell transformation assay

Cells were seeded at 8 × 10^3^ cells/per well into 6-well plates and cultured in 1ml of 0.33% BME agar containing 10% FBS, with an additional 3ml of 0.5% BME agar containing 10% FBS below. After the cells were cultured in a 37°C, 5% CO_2_ incubator for 5–10 days, at which time colonies were observed by microscopy.

### Statistical analysis

All quantitative experiments were performed in triplicate at minimum. Statistical analysis was performed using Graphpad prism. Student's *t*-test was used to evaluate the data. Times for OS were defined from treatment initiation to date of death or last follow-up. The correlation between the level of TOPK or Ki67 and OS were assessed by log-rank test. In all tests, differences were considered significant at *P* < 0.05.

## References

[1] Hou L, Jiang J, Liu B, Han W, Wu Y, Zou X, Nasca PC, Xue F, Chen Y, Zhang B, Pang H, Wang Y, Wang Z, Li J (2016). Smoking and adult glioma: a population-based case-control study in China. Neuro-oncol.

[2] Louis DN, Perry A, Reifenberger G, von Deimling A, Figarella-Branger D, Cavenee WK, Ohgaki H, Wiestler OD, Kleihues P, Ellison DW (2016). The 2016 World Health Organization Classification of Tumors of the Central Nervous System: a summary. Acta Neuropathol.

[3] Zhu F, Zykova TA, Kang BS, Wang Z, Ebeling MC, Abe Y, Ma WY, Bode AM, Dong Z (2007). Bidirectional signals transduced by TOPK-ERK interaction increase tumorigenesis of HCT 116 colorectal cancer cells. Gastroenterology.

[4] Hu F, Gartenhaus RB, Zhao XF, Fang HB, Minkove S, Poss DE, Rapoport AP (2013). c-Myc and E2F1 drive PBK/TOPK expression in high-grade malignant lymphomas. Leuk Res.

[5] Zykova TA, Zhu F, Vakorina TI, Zhang J, Higgins LA, Urusova DV, Bode AM, Dong Z (2010). T-LAK cell-originated protein kinase (TOPK) phosphorylation of Prx1 at Ser-32 prevents UVB-induced apoptosis in RPMI7951 melanoma cells through the regulation of Prx1 peroxidase activity. J Biol Chem.

[6] Park JH, Lin ML, Nishidate T, Nakamura Y, Katagiri T (2006). PDZ-binding kinase/T-LAK cell-originated protein kinase, a putative cancer/testis antigen with an oncogenic activity in breast cancer. Cancer Res.

[7] Lei B, Qi W, Zhao Y, Li Y, Liu S, Xu X, Zhi C, Wan L, Shen H (2015). PBK/TOPK expression correlates with mutant p53 and affects patients’ prognosis and cell proliferation and viability in lung adenocarcinoma. Hum Pathol.

[8] He F, Yan Q, Fan L, Liu Y, Cui J, Wang J, Wang L, Wang Y, Wang Z, Guo Y, Huang G (2010). PBK/TOPK in the differential diagnosis of cholangiocarcinoma from hepatocellular carcinoma and its involvement in prognosis of human cholangiocarcinoma. Hum Pathol.

[9] Gaudet S, Branton D, Lue RA (2000). Characterization of PDZ-binding kinase, a mitotic kinase. Proc Natl Acad Sci U S A.

[10] Joel M, Mughal AA, Grieg Z, Murrell W, Palmero S, Mikkelsen B, Fjerdingstad HB, Sandberg CJ, Behnan J, Glover JC, Langmoen IA, Stangeland B (2015). Targeting PBK/TOPK decreases growth and survival of glioma initiating cells in vitro and attenuates tumor growth in vivo. Mol Cancer.

[11] Ayllón V, O'connor R (2007). PBK/ TOPK promotes tumour cell proliferation through p38 MAPK activity and regulation of the DNA damage response. Oncogene.

[12] Zlobec I, Molinari F, Kovac M, Bihl MP, Altermatt HJ, Diebold J, Frick H, Germer M, Horcic M, Montani M, Singer G, Yurtsever H, Zettl A (2010). Prognostic and predictive value of TOPK stratified by KRAS and BRAF gene alterations in sporadic, hereditary and metastatic colorectal cancer patients. Brit J Cancer.

[13] Lei B, Liu S, Qi W, Zhao Y, Li Y, Lin N, Xu X, Zhi C, Mei J, Yan Z, Wan L, Shen H (2013). PBK/TOPKexpression in non-small-cell lung cancer: its correlation and prognostic significance with Ki67 and p53 expression. Histopathology.

[14] Ikeda Y, Park JH, Miyamoto T, Takamatsu N, Kato T, Iwasa A, Okabe S, Imai Y, Fujiwara K, Nakamura Y, Hasegawa K (2016). T-LAK cell-originated protein kinase (TOPK) as a prognostic factor and a potential therapeutic target in ovarian cancer. Clin Cancer Res.

[15] He H, Yao M, Zhang W, Tao B, Liu F, Li S, Dong Y, Zhang C, Meng Y, Li Y, Hu G, Luo C, Zong H, Lu Y (2016). MEK2 is a prognostic marker and potential chemo-sensitizing target for glioma patients undergoing temozolomide treatment. Cell Mol Immunol.

[16] Zhang J, Stevens MF, Bradshaw TD (2012). Temozolomide: mechanisms of action, repair and resistance. Curr Mol Pharmacol.

[17] Fan CH, Liu WL, Cao H, Wen C, Chen L, Jiang G (2013). O6-methylguanine DNA methyltransferase as a promising target for the treatment of temozolomide-resistant gliomas. Cell Death Dis.

[18] Esteller M, Garcia-Foncillas J, Andion E, Goodman SN, Hidalgo OF, Vanaclocha V, Baylin SB, Herman J (2000). Inactivation of the DNA-repair gene MGMT and the clinical response of gliomas to alkylating agents. N Engl J Med.

[19] Zhang J, Stevens MF, Hummersone M, Madhusudan S, Laughton CA, Bradshaw TD (2011). Certain imidazotetrazines escape O6-methylguanine-DNA methyltransferase and mismatch repair. Oncology.

[20] Montaldi AP, Godoy PR, Sakamoto-Hojo ET (2015). APE1/REF-1 down-regulation enhances the cytotoxic effects of temozolomide in a resistant glioblastoma cell line. Mutat Res Genet Toxicol Environ Mutagen.

[21] Hu X, Miao W, Zou Y, Zhang W, Zhang Y, Liu H (2013). Expression of p53, epidermal growth factor receptor, Ki-67 and O6 methylguanine-DNA methyltransferase in human gliomas. Oncol Lett.

[22] Nagane M, Levitzki A, Gazit A, Cavenee WK, Huang HJ (1998). Drug resistance of human glioblastoma cells conferred by a tumor-specific mutant epidermal growth factor receptor through modulation of Bcl-XL and caspase-3-like proteases. Proc Natl Acad Sci U S A.

[23] Otani R, Uzuka T, Ueki K (2017). Classification of adult diffuse gliomas by molecular markers-a short review with historical footnote. Jpn J Clin Oncol.

[24] Kim DJ, Li Y, Reddy K, Lee MH, Kim MO, Cho YY, Lee SY, Kim JE, Bode AM, Dong Z (2012). Novel PBK/TOPK inhibitor HI-PBK/TOPK-032 effectively suppresses colon cancer growth. Cancer Res.

[25] Balvers RK, Lamfers ML, Kloezeman JJ, Kleijn A, Berghauser Pont LM, Dirven CM, Leenstra S (2015). ABT-888 enhances cytotoxic effects of temozolomide independent of MGMT status in serum free cultured glioma cells. J Transl Med.

[26] Banáth JP, Klokov D, MacPhail SH, Banuelos CA, Olive PL (2010). Residual gammaH2AX foci as an indication of lethal DNA lesions. BMC Cancer.

[27] Yan H, Parsons DW, Jin G, McLendon R, Rasheed BA, Yuan W, Kos I, Batinic-Haberle I, Jones S, Riggins GJ, Friedman H, Friedman A, Reardon D (2009). IDH1 and IDH2 Mutations in Gliomas. N Engl J Med.

[28] Liu Y, Hu H, Zhang C, Wang Z, Li M, Jiang T (2016). Integrated analysis identified genes associated with a favorable prognosis in oligodendrogliomas. Genes Chromosomes Cancer.

[29] Wong AJ, Ruppert JM, Bigner SH, Grzeschik CH, Humphrey PA, Bigner DS, Vogelstein B (1992). Structural alterations of the epidermal growth factor receptor gene in human gliomas. Proc Natl Acad Sci U S A.

[30] Gao YF, Mao XY, Zhu T, Mao CX, Liu ZX, Wang ZB, Li L, Li X, Yin JY, Zhang W, Zhou HH, Liu ZQ (2016). COL3A1 and SNAP91: novel glioblastoma markers with diagnostic and prognostic value. Oncotarget.

[31] Tsai WC, Hueng DY, Nieh S, Gao HW (2017). ARID4B is a good biomarker to predict tumour behaviour and decide WHO grades in gliomas and meningiomas. J Clin Pathol.

[32] Montine TJ, Vandersteenhoven JJ, Aguzzi A, Boyko OB, Dodge RK, Kerns BJ, Burger PC (1994). Prognostic significance of Ki67 proliferation index in supratentorial fibrillary astrocytic neoplasms. Neurosurgery.

[33] Zeng X, Liu L, Zheng M, Sun H, Xiao J, Lu T, Huang G, Chen P, Zhang J, Zhu F, Li H, Duan Q (2016). Pantoprazole, an FDA-approved proton-pump inhibitor, suppresses colorectal cancer growth by targeting T-cell-originated protein kinase. Oncotarget.

[34] Zykova TA, Zhu F, Wang L, Li H, Bai R, Lim DY, Yao K, Bode AM, Dong Z (2017). The T-LAK Cell-originated Protein Kinase Signal Pathway Promotes Colorectal Cancer Metastasis. EBioMedicine.

[35] Seol MA, Park JH, Jeong JH, Lyu J, Han SY, Oh SM (2017). Role of TOPK in lipopolysaccharide-induced breast cancer cell migration and invasion. Oncotarget.

[36] Park JH, Inoue H, Kato T, Zewde M, Miyamoto T, Matsuo Y, Salgia R, Nakamura Y (2017). TOPK (T-LAK cell-originated protein kinase) inhibitor exhibits growth suppressive effect on small cell lung cancer. Cancer Sci.

[37] Ohashi T, Komatsu S, Ichikawa D, Miyamae M, Okajima W, Imamura T, Kiuchi J, Nishibeppu K, Kosuga T, Konishi H, Shiozaki A, Fujiwara H, Okamoto K (2016). Overexpression of PBK/TOPK Contributes to Tumor Development and Poor Outcome of Esophageal Squamous Cell Carcinoma. Anticancer Res.

[38] Ohashi T, Komatsu S, Ichikawa D, Miyamae M, Okajima W, Imamura T, Kiuchi J, Kosuga T, Konishi H, Shiozaki A, Fujiwara H, Okamoto K, Tsuda H (2017). Overexpression of PBK/TOPK relates to tumour malignant potential and poor outcome of gastric carcinoma. Br J Cancer.

[39] Hu F, Gartenhaus RB, Eichberg D, Liu Z, Fang HB, Rapoport AP (2010). PBK/TOPK interacts with the DBD domain of tumor suppressor p53 and modulates expression of transcriptional targets including p21. Oncogene.

[40] Shih MC, Chen JY, Wu YC, Jan YH, Yang BM, Lu PJ, Cheng HC, Huang MS, Yang CJ, Hsiao M, Lai JM (2012). TOPK/PBK promotes cell migration via modulation of the PI3K/PTEN/AKT pathway and is associated with poor prognosis in lung cancer. Oncogene.

[41] Wei DC, Yeh YC, Hung JJ, Chou TY, Wu YC, Lu PJ, Cheng HC, Hsu YL, Kuo YL, Chen KY, Lai JM (2012). Overexpression of T-LAK cell-originated protein kinase predicts poor prognosis in patients with stage I lung adenocarcinoma. Cancer Sci.

[42] Murat A, Migliavacca E, Gorlia T, Lambiv WL, Shay T, Hamou MF, de Tribolet N, Regli L, Wick W, Kouwenhoven MC, Hainfellner JA, Heppner FL, Dietrich PY (2008). Stem cell-related “self-renewal” signature and high epidermal growth factor receptor expression associated with resistance to concomitant chemoradiotherapy in glioblastoma. J Clin Oncol.

[43] Park JH, Yoon DS, Choi HJ, Hahm DH, Oh SM (2013). Phosphorylation of IκBα at serine 32 by T-lymphokine-activated killer cell-originated protein kinase is essential for chemoresistance against doxorubicin in cervical cancer cells. J Biol Chem.

[44] Lan F, Yang Y, Han J, Wu Q, Yu H, Yue X (2016). Sulforaphane reverses chemoresistance to temozolomide in glioblastoma cells by NF-κB-dependent pathway downregulating MGMT expression. Int J Oncol.

[45] Nagane M, Asai A, Shibui S, Nomura K, Matsutani M, Kuchino Y (1992). Expression of O6-methylguanine-DNA methyltransferase and chloroethylnitrosourea resistance of human brain tumors. Jpn J Clin Oncol.

[46] Zykova TA, Zhu F, Lu C, Higgins L, Tatsumi Y, Abe Y, Bode AM, Dong Z (2006). Lymphokine-activated killer T-cell-originated protein kinase phosphorylation of histone H2AX prevents arsenite-induced apoptosis in RPMI7951 melanoma cells. Clin Cancer Res.

[47] Kang NJ, Lee KW, Kim BH, Bode AM, Lee HJ, Heo YS, Boardman L, Limburg P, Lee HJ, Dong Z (2011). Coffee phenolic phytochemicals suppress colon cancer metastasis by targeting MEK and TOPK. Carcinogenesis.

[48] Matsuo Y, Park JH, Miyamoto T, Yamamoto S, Hisada S, Alachkar H, Nakamura Y (2014). TOPK inhibitor induces complete tumor regression in xenograft models of human cancer through inhibition of cytokinesis. Sci Transl Med.

[49] Yuan J, Gu K, He J, Sharma S (2013). Preferential up-regulation of osteopontin in primary central nervous system lymphoma does not correlate with putative receptor CD44v6 or CD44H expression. Hum Pathol.

[50] Montgomery RM, Queiroz LS, Rogerio F (2015). EGFR, p53, IDH-1 and MDM2 immunohistochemical analysis in glioblastoma: therapeutic and prognostic correlation. Arq Neuropsiquiatr.

[51] Moffat J, Grueneberg DA, Yang X, Kim SY, Kloepfer AM, Hinkle G, Piqani B, Eisenhaure TM, Luo B, Grenier JK, Carpenter AE, Foo SY, Stewart SA (2006). A lentiviral RNAi library for human and mouse genes applied to an arrayed viral high-content screen. Cell.

